# Evidence of SARS-CoV-2-Specific T-Cell-Mediated Myocarditis in a MIS-A Case

**DOI:** 10.3389/fimmu.2021.779026

**Published:** 2021-12-09

**Authors:** Kevin M. Vannella, Cihan Oguz, Sydney R. Stein, Stefania Pittaluga, Esra Dikoglu, Arjun Kanwal, Sabrina C. Ramelli, Thomas Briese, Ling Su, Xiaolin Wu, Marcos J. Ramos-Benitez, Luis J. Perez-Valencia, Ashley Babyak, Nu Ri Cha, Joon-Yong Chung, Kris Ylaya, Ronson J. Madathil, Kapil K. Saharia, Thomas M. Scalea, Quincy K. Tran, Daniel L. Herr, David E. Kleiner, Stephen M. Hewitt, Luigi D. Notarangelo, Alison Grazioli, Daniel S. Chertow

**Affiliations:** ^1^ Emerging Pathogens Section, Critical Care Medicine Department, Clinical Center, National Institutes of Health, Bethesda, MD, United States; ^2^ Laboratory of Immunoregulation, National Institute of Allergy and Infectious Diseases, National Institutes of Health, Bethesda, MD, United States; ^3^ National Institute of Allergy and Infectious Diseases Collaborative Bioinformatics Resource, Frederick National Laboratory for Cancer Research, Leidos Biomedical Research, Inc., Frederick, MD, United States; ^4^ Advanced Biomedical Computational Science, Frederick National Laboratory for Cancer Research, Leidos Biomedical Research, Inc., Frederick, MD, United States; ^5^ Laboratory of Pathology, Center for Cancer Research, National Cancer Institute, National Institutes of Health, Bethesda, MD, United States; ^6^ Division of Cardiology, Westchester Medical Center, Valhalla, NY, United States; ^7^ Critical Care Medicine Department, Clinical Center, National Institutes of Health, Bethesda, MD, United States; ^8^ Center for Infection and Immunity, Columbia University Mailman School of Public Health, New York, NY, United States; ^9^ Cancer Research Technology Program, Frederick National Laboratory for Cancer Research, Leidos Biomedical Research, Inc., Frederick, MD, United States; ^10^ Postdoctoral Research Associate Training Program, National Institute of General Medical Sciences, National Institutes of Health, Bethesda, MD, United States; ^11^ Department of Transfusion Medicine, Clinical Center, National Institutes of Health, Bethesda, MD, United States; ^12^ Department of Surgery, Division of Cardiac Surgery, University of Maryland School of Medicine, Baltimore, MD, United States; ^13^ Department of Medicine, Division of Infectious Disease, University of Maryland School of Medicine, Baltimore, MD, United States; ^14^ Department of Surgery, Program in Trauma, R. Adams Cowley Shock Trauma Center, University of Maryland School of Medicine, Baltimore, MD, United States; ^15^ Department of Emergency Medicine, R. Adams Cowley Shock Trauma Center, University of Maryland School of Medicine, Baltimore, MD, United States; ^16^ Department of Medicine, Program in Trauma, R. Adams Cowley Shock Trauma Center, University of Maryland School of Medicine, Baltimore, MD, United States; ^17^ Laboratory of Clinical Immunology and Microbiology, National Institute of Allergy and Infectious Diseases, National Institutes of Health, Bethesda, MD, United States; ^18^ Kidney Diseases Branch, Kidney Disease Section, National Institute of Diabetes and Digestive and Kidney Diseases, National Institutes of Health, Bethesda, MD, United States

**Keywords:** MIS-A, myocarditis, SARS-CoV-2, T cell receptor (TCR), SARS-CoV-2 epitopes, CDR3 sequences, cd-hit

## Abstract

A 26-year-old otherwise healthy man died of fulminant myocarditis. Nasopharyngeal specimens collected premortem tested negative for severe acute respiratory syndrome coronavirus 2 (SARS-CoV-2). Histopathological evaluation of the heart showed myocardial necrosis surrounded by cytotoxic T-cells and tissue-repair macrophages. Myocardial T-cell receptor (TCR) sequencing revealed hyper-dominant clones with highly similar sequences to TCRs that are specific for SARS-CoV-2 epitopes. SARS-CoV-2 RNA was detected in the gut, supporting a diagnosis of multisystem inflammatory syndrome in adults (MIS-A). Molecular targets of MIS-associated inflammation are not known. Our data indicate that SARS-CoV-2 antigens selected high-frequency T-cell clones that mediated fatal myocarditis.

## Introduction

In the weeks after SARS-CoV-2 infection, some individuals develop an emerging and life-threatening hyperinflammatory illness called multisystem inflammatory syndrome (MIS) in children (C) or adults (A) (1–3). Most reports of MIS-C (86.5%) (1) and MIS-A (82.4%) (3) describe cardiovascular involvement. Cardiac manifestations are frequently severe and include electrocardiogram and cardiac magnetic resonance abnormalities along with elevated circulating troponin and left ventricular impairment indicative of acute myocarditis (1–5).

Diagnosis of MIS-C and MIS-A requires confirmation of previous SARS-CoV-2 infection or a recent known exposure (1, 2). Although MIS-C/A is thought to represent a postinfectious immune-mediated process, it is unknown whether the target of inflammatory cells is SARS-CoV-2 or host antigens.

Here, we present the hospital course and postmortem analysis of a previously healthy 26-year-old patient whose fulminant myocarditis in May 2020, raised suspicion of Covid-19 despite negative SARS-CoV-2 testing premortem. Postmortem, we detected SARS-CoV-2 RNA in the duodenum, ileum, and appendix amongst 29 tissues sampled. Although we did not detect RNA in the heart, we found compelling evidence for hyper-dominant SARS-CoV-2-specific T-cells in the myocardial infiltrate. These data suggest that in this case of MIS-A, prior SARS-CoV-2 infection resulted in fatal myocarditis driven by SARS-CoV-2-specific T-cells.

## Materials and Methods

### Autopsy

Autopsies were performed and tissues were collected as previously described (6) in the National Cancer Institute’s Laboratory of Pathology at the National Institutes of Health Clinical Center following consent of the legal next of kin.

### SARS-CoV-2 RNA Detection by RNAscope

RNAscope was performed as previously described at the NIH (6) except 3,3’-diaminobenzidine (DAB) was used as a chromogen.

### SARS-CoV-2 RNA Detection by ddPCR

RNAlater (Invitrogen)-preserved autopsy tissues were homogenized and processed as previously described (6). Total RNA was extracted using RNeasy Mini, RNeasy Fibrous Tissue Mini, and RNeasy Lipid Tissue Mini Kits (Qiagen). The QX200 AutoDG Droplet Digital PCR (ddPCR) System (Bio-Rad) was used to detect SARS-CoV-2 RNA using the SARS-CoV-2 ddPCR Kit (Bio-Rad) (6). For samples to be considered positive for SARS-CoV-2 nucleocapsid 1 (N1) or 2 (N2) genes, the manufacturer’s limit of detection of >/= 0.1 copies/µl final ddPCR reaction and 2 positive droplets per well was required.

### Immunohistochemistry

We performed immunohistochemical stains on formalin-fixed paraffin-embedded three micron tissue sections using an automated immunostainer Benchmark Ultra (Roche) with an Ultra-View Universal DAB brown detection kit (alongside an Ultra-View Universal AP Red detection kit for double staining). These antibodies were used for the following targets: CD3 (clone 2GV6, predilute Roche Cat # 790-4341), CD4 (clone SP35, predilute Roche Cat# 790-4423), CD8 (clone SP57, predilute Roche Cat#790-4460), granzyme B (clone GrB-7+D170, dilution 1:100, Millipore Cat# MAB 3070), perforin (clone KM585 PI-8) (dilution 1:200, Leica Cat# NCL-L-perforin) detection with Optiview kit (Roche), CD68 (clone KP1, predilute Roche Cat#790-2931), CD163 (clone MRQ-26, predilute Roche Cat#760-4437).

### VirCapSeq

cDNA preparations were processed for viral capture high-throughput sequencing (VirCapSeq-VERT (7)) with an iScript cDNA synthesis kit (Bio-Rad). Library construction used Kapa HyperPlus kits (Roche) with enzymatic fragmentation to 300 bp average size. Individually dual-barcoded libraries were pooled and 150 cycle single-pass reads generated (Ilumina NextSeq 550). Per barcode, 7 – 10 million raw reads were obtained. Fastq files were adapter trimmed (Cutadapt v 3.0) (8) and quality reports generated (FastQC v 0.11.5) (9). Reads were quality filtered and end trimmed with PRINSEQ (v 0.20.3) (10). Host reads were removed by mapping against a human reference database using Bowtie2 (11). The remaining reads were subjected to homology search using NCBI Megablast against the viral GenBank nucleotide database. The blast reports were annotated with taxonomic information to assess read frequencies for viral families, genera, species and individual GenBank sequence accession numbers. Human endogenous retroviruses served as positive technical controls.

### T-Cell Receptor-Epitope Analysis

Genomic DNA was extracted from formalin-fixed paraffin-embedded (FFPE) tissue using a QIAamp DNA FFPE kit. Complementarity determining regions (CDR)3 of T-cell receptor (TCR)β chains present in the human tissue samples were sequenced in a high-throughput manner using the immunoSEQ assay (12) after amplification of the extracted DNA in a bias-controlled multiplex PCR. The resulting CDR3 sequences were collapsed and filtered to quantify the absolute abundance and frequency of each unique TCRβ CDR3 region with Adaptive Biotechnologies’ pipeline (13). We used CD-HIT (14) to test whether the most frequently observed CDR3 sequences derived from the patient samples clustered with the SARS-CoV-2-specific TCR sequences from ImmuneCODE (13). We generated the clonality and repertoire overlap figure using Immunarch (15).

### HLA Typing

Typing was performed as previously described (16).

## Results

### Clinical History

A previously healthy 26-year-old man presented to the hospital with complaints of chest pressure and shortness of breath for 1 week and nausea, vomiting, and chills for 3 days. He was afebrile, had a heart rate of 80 beats per minute, and was normotensive with an unremarkable physical examination. SARS-CoV-2 PCR testing of a nasopharyngeal (NP) swab specimen was negative. Basic metabolic panel and complete blood count values were within normal range although troponin I and C-reactive protein were elevated to 21.2 ng/ml (ref: 0-0.045) and 9.84 mg/L (ref: 0-3), respectively, indicative of myocardial injury and inflammation. Electrocardiogram demonstrated inferolateral ST changes concerning for myocardial ischemia (**Figure S1A**). Chest X-Ray showed no abnormalities (**Figure S2**). The patient was admitted to the medical ward for non-ST segment elevation myocardial infarction with plans for coronary angiogram in the morning.

The following morning, a transthoracic echocardiogram demonstrated an ejection fraction (EF) of 60-65%, without wall motion or valvular abnormalities. Coronary angiogram demonstrated mild non-obstructive coronary artery disease (**Figure S3**) and left ventriculogram showed intact function without regional wall motion abnormalities or mitral regurgitation. A repeat NP swab for SARS-CoV-2 was negative. Post-cardiac catheterization he experienced ongoing chest pain with rising troponin I levels (43.7 ng/ml), and his electrocardiogram was unchanged. Colchicine and ibuprofen were initiated for presumptive myopericarditis.

In the setting of recurrent chest pain, an electrocardiogram demonstrated persistent ST segment changes concerning for ischemia (**Figure S1B**). Repeat troponin I level was 83.5 ng/ml, and lactate was 3.0 mmol/L (ref: 0.7-2.0). He developed sustained ventricular tachycardia (VT) progressing to pulseless VT arrest. Return of spontaneous circulation was achieved. However, post-arrest point-of care echocardiogram showed global left ventricular hypokinesis with an estimated EF <10%. He was started on an epinephrine infusion and cannulated for veno-arterial extracorporeal membrane oxygenation (ECMO) by cardiothoracic surgery at bedside. He was transferred to a tertiary care ECMO facility, where his NP sample again tested negative for SARS-CoV-2. Shortly after arrival he suffered a pulseless electrical activity arrest with profound vasodilation and died.

### Postmortem Analysis

**Figure 1:**
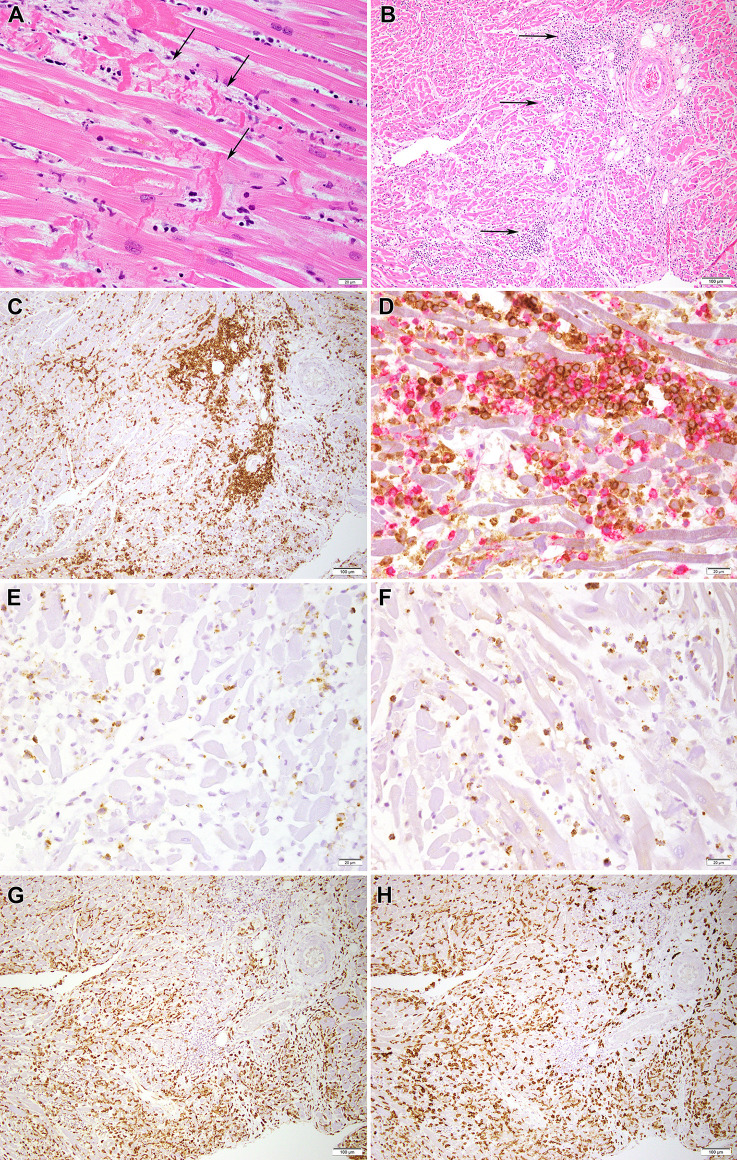
Histopathology images of myocardium. **(A)** H&E section from the myocardium shows contraction band myocyte necrosis (arrows) (40x). **(B)** H&E section from the myocardium shows an inflammatory infiltrate mostly composed of lymphocytes and macrophages (arrows) (10x) as seen better in **(C)** by an immunohistochemical stain for CD3, which marks T lymphocytes (10x). **(D)** A double immunohistochemical stain highlights the presence of CD8-positive T cells (red chromogen) and CD4-positive T cells (brown chromogen); low grade expression by CD4 marks macrophages (40x); **(E, F)** show the presence of scattered cytotoxic T cells positive for perforin (40X) and granzyme B (40X), respectively. **(G, H)** Presence of numerous histiocytes/macrophages is confirmed by CD68 (10x) and CD163 (10x) staining, respectively.

Cardiac histopathological findings featured microscopic foci of contraction band myocyte necrosis (**Figure 1A**) and extensive lymphocytic infiltration reflecting severe myocarditis (**Figure 1B**). The lymphocytic infiltrate colocalized with dying myocytes and was more extensive in the right ventricle (RV) than the left ventricle (LV). Immunohistochemical studies were performed on a section from the RV. The vast majority of lymphocytes were T-cells (**Figure 1C**). Both CD4+ and CD8+ T-cells were present (**Figure 1D**). CD8+ cells were positive for perforin (**Figure 1E**), and granzyme B (**Figure 1F**). Only rare CD20+ B cells and no CD56+ natural killer cells were observed. Macrophages staining with CD68 (**Figure 1G**) and CD163 (marker of M2 tissue repair phenotype; **Figure 1H**) outnumbered CD3+ T-cells.

Extracardiac findings included mild pneumonia with pulmonary edema and mild interstitial pneumonitis. Sinusoidal histiocytosis of pulmonary hilar lymph nodes was observed, and lymphoid depletion was noted in the spleen.

Assays for SARS-CoV-2 RNA in cardiac tissues by RNAscope and ddPCR were negative and below the limit of detection, respectively. However, we detected SARS-CoV-2 RNA in the mucosal epithelium of the patient’s appendix (**Figure S4**) and duodenum by RNAscope, and in the ileum by ddPCR (**Table S1**). We used VirCapSeq-VERT to test for the presence of other viruses in myocardial and pericardial tissues and found none (**Table S2**).

**Figure 2:**
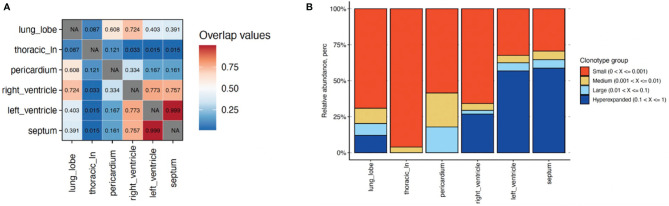
Characterization of T cell clonotype abundance in cardiac tissues alongside lung and lymph node for comparison. **(A)** T cell repertoire overlap across tissues using Morisita indices (1&red=highest; 0&blue=lowest). **(B)** Percentage of T cell clonotypes with small (0-0.001, orange); medium (0.001-0.01, tan); large (0.01-0.1; light blue); and hyperexpanded (0.1-1; dark blue) frequencies across tissues. NA, Not applicable.

We sequenced TCRs in cardiac tissues and found a high degree of TCR repertoire overlap across RV, LV, and intraventricular septum (**Figure 2A**). We observed three hyperexpanded clones across these tissues (**Table S3**; **Figure 2B**). The most dominant clonotype (clonotype1) comprised 27-59% of T-cells across tissues, while clonotypes 2 and 3 comprised 2.5-4.3% and 1.0-1.7% of T-cells, respectively (**Table S3**).

An epitope is often recognized by many distinct TCRs with highly similar CDR3 sequences (17). Therefore, we applied a widely-used CD-HIT clustering approach (14) to assess whether the CDR3 sequences of clonotypes1-3 (**Table S4**) were highly similar to the 149,845 unique TCRβ CDR3 sequences that have been verified to bind to SARS-CoV-2 epitopes and catalogued in ImmuneCODE (13). Clonotypes1-3 had nine, one, and two matches with >90% amino acid sequence identity to TCRs in ImmuneCODE, respectively (**Table 1**). No CDR3 sequences from clonotypes with a frequency <1% in myocardial tissues had an ImmuneCODE hit.

**Table 1:** Similarity-based matches of TCR sequences from patient’s dominant clonotypes with validated SARS-CoV-2-specific TCR sequences.

Seq ID	CDR3 AASeq	Global sequence identity (%)	Levenshtein distance	Seq source
**Clonotypel**	**CASSYSSGFTDTQYF**	NA	NA	Patient
P18688	CASSYSSTDTQYF	100	2	lmmuneCODE
P19422	CASSYSSGGTDTQYF	93.33	1	lmmuneCODE
P26312	CASSFSGFTDTQYF	92.86	2	lmmuneCODE
P39473	CASSESSGFTDTQYF	93.33	1	lmmuneCODE
P73013	CASSYSTFTDTQYF	92.86	2	lmmuneCODE
P73132	CASSYSSVTDTQYF	92.86	2	lmmuneCODE
P97143	CASSYSGFTDTQYF	100	1	lmmuneCODE
P118904	CASSYSSPTDTQYF	92.86	2	lmmuneCODE
P120946	CASSYSPGFTDTQYF	93.33	1	lmmuneCODE
**Clonotype2**	**CASSRLAGRETQYF**	NA	NA	Patient
P137116	CASSRLAGREQYF	100	1	lmmuneCODE
**Clonotype3**	**CASSDSLLNQPQHF**	NA	NA	Patient
P47858	CASSDSLNQPQHF	100	1	lmmuneCODE
P119742	CASSLLNQPQHF	100	2	lmmuneCODE

CD-HIT-derived clusters that contain at least one CDR3 amino acid sequence from a dominant clonotype (1-3) as well as one ImmuneCode-based SARS-CoV-2-specific CDR3 sequence. The clustering threshold was 90% global sequence identity. Global sequence identity was defined by CD-HIT as the number of identical amino acids in alignment divided by the number of amino acids in the shorter sequence, allowing for a sequence length difference of up to two amino acids per alignment. Levenshtein distances between the patient clonotypes and the ImmuneCODE hits were computed using the R stringdist package. Levenshtein distance is defined as the minimum number of amino acid edits (insertions, deletions or substitutions) needed to change one amino acid sequence into the other. NA, Not applicable.

The CDR3 sequences clustered with clonotype1 have been shown to bind 15 unique epitopes from three SARS-CoV-2 antigens (ORF1ab, spike, ORF7b) (**Table S5**). CDR3 sequences clustered with clonotypes2 and 3 have been shown to bind 19 unique SARS-CoV-2 epitopes from ORF10, ORF1ab, and ORF7b. The Multiplex Identification of Antigen-Specific T-Cell Receptors Assay (MIRA), used to define the list of epitopes in ImmuneCODE (13), screens for a finite set of epitopes per unique TCR sequence. Given this, we queried The Immune Epitope Database (IEDB) and identified an additional 26 well-characterized SARS-CoV-2 epitopes that shared >90% amino acid sequence similarity with ImmuneCODE epitopes (**Table S6**) (18).

Lastly, to identify possible cross-reactive human epitopes that could exacerbate the patient’s T-cell infiltrate, we queried whether there were human epitopes that matched the ImmuneCODE SARS-CoV-2 epitopes with >90% amino acid similarity. We found seven human epitope matches (**Table S7**). Three of these epitopes originate from plectin, a cytoskeletal protein that maintains tissue integrity and has been previously associated with myocarditis and dilated cardiomyopathy (19).

## Discussion

There is limited information on mechanisms of MIS pathogenesis. While clinical observations suggest involvement of the innate and/or adaptive immune responses, the potential contribution of the cellular and humoral components remains ill-defined (20). Possible mechanisms include antibody or T-cell-mediated recognition of SARS-CoV-2 antigens expressed on infected cells, cross-reactivity of SARS-CoV-2 epitopes with tissue-specific self-antigens, virus-encoded superantigens driving T-cell activation, and formation of immune complexes that promote inflammation.

Here, we present evidence that T-cells specific for SARS-CoV-2 antigens contributed to fatal myocarditis in a patient with MIS-A. In addition to cardiac dysfunction, the patient’s laboratory results indicated severe inflammation in the absence of significant respiratory illness. The patient did not have blood available at the time of our postmortem studies for serology. Instead, we confirmed that the patient was infected with SARS-CoV-2 by detecting SARS-CoV-2 RNA in gut tissues *via* RNAscope and ddPCR.

The relevance of SARS-CoV-2 presence in the gut to the development of MIS-A is not yet clear. However, a recent study of MIS-C patients indicated that prolonged presence of SARS-CoV-2 in the gut increased intestinal permeability (21). The authors proposed that the permeability led to leakage of SARS-CoV-2 antigens into the blood and subsequent hyperinflammation. More research is required to determine whether this is an essential causative mechanism of MIS-C or MIS-A pathogenesis, but these data offer a clue about the possible etiologic relevance of finding SARS-CoV-2 only in the gut in this case of MIS-A.

Several pieces of evidence from our CDR3 clustering analysis support that the three highest frequency T-cell clones in the heart were specific for SARS-CoV-2. Clonotype1 comprised about half of the patient’s myocardial T-cells and expressed highly similar TCRβ CDR3 sequences to nine TCRs that have been shown to bind SARS-CoV-2 epitopes. The CDR3 sequences of clonotype1 were also associated with five different V genes (**Table S4**) indicating that immunodominance of clonotype1 was likely the result of viral antigen-driven convergent recombination. Furthermore, across the patient’s cardiac T-cell repertoire, the three highest frequency clones also had the highest number of ImmuneCODE hits.

While the CDR3 clustering approaches we applied identify similar CDR3 sequences with common specificity to antigenic peptides (22), they are limited to in silico predictive power. Confirmatory *in vitro* functional testing could not be accomplished in this case as viable T-cell clones were unavailable.

It remains unclear why the SARS-CoV-2-specific T-cells would traffic preferentially to heart tissues as opposed to other tissues particularly when we could not detect SARS-CoV-2 there at the time of autopsy. Given that MIS often manifests weeks after SARS-CoV-2 infection, it is possible that SARS-CoV-2 was previously present in the heart but was cleared by the time of death. The hypothesis that death occurred at a later phase of myocarditis is supported by the predominance of CD163+ wound-healing macrophages that colocalized with the T-cells and damaged myocytes at the time of autopsy. The frequency of identification of infectious agents with infectious heart disease varies in part due to the timing of sample collection (23, 24). Cardiotropic coxsackievirus B3 is an example of a virus that has been shown to initiate myocarditis through damage to the heart tissue despite leaving little evidence of replicating virus behind (25–27).

Virus-induced injury could lead to the exposure or release of self-antigens that lead to autoimmune myocarditis (25). Our findings suggest that the T-cell response in our case is directed predominantly against SARS-CoV-2 and not self-antigens, but they do not rule out the possibility that a self-antigen could have promoted myocarditis in the absence of cardiac infection or after cardiac infection through viral mimicry. Such a mechanism has been described with bacterial mimicry. Heart-specific T-cells can be primed in the gut by the commensal *Bacteroides* that contains peptides that mimic cardiac myosin heavy chain peptides (28). After an inflammatory trigger such as viral infection, these cardiac myosin-specific T cells can mediate myocarditis in genetically susceptible individuals with HLA variants that bind these peptides. The SARS-CoV-2 epitopes that we identified with sequences similar to human epitopes (**Table S7**) have previously identified HLA restrictions (29) that closely match the HLA types of the patient (**Table S8**). While none of the human epitopes we identified were definitively cardiac-specific, the consistency of HLA restrictions increases the possibility that cross-reactivity could have facilitated preferential homing of antigen-specific T-cells in this case.

In conclusion, T-cells targeting SARS-CoV-2 appear to be central to the fatal myocarditis in this case. This report provides initial evidence that the cellular host immune response directed at SARS-CoV-2 led to severe cardiac dysfunction and death in a case of MIS-A. TCR sequencing and identification of corresponding epitopes is a useful strategy for understanding mechanisms of organ injury in MIS and other disorders that cause pathogenic host immune cell infiltrates in tissues.

## Data Availability Statement

The datasets presented in this study can be found in online repositories. The names of the repository/repositories and accession number(s) can be found below: https://clients.adaptivebiotech.com/pub/vannella-2021-fi.

## Author Contributions

DC conceived the study. DC and KV supervised the study. CO and LN devised the bioinformatics approach. AK, RM, KS, TS, QT, DH, AG managed or collected clinical data. KV, CO, SS, SP, ED, SR, TB, LS, XW, MR, LP, AB, NC, JC, KY, DK, SH collected or analyzed postmortem data. KV wrote the manuscript. All authors reviewed and edited the manuscript and approved the final version.

## Funding

This study was funded and supported by the Intramural Research Program of the NIH, Clinical Center and National Institute of Allergy and Infectious Diseases.

## Conflict of Interest

Authors CO, LS and XW were employed by company Leidos Biomedical Research, Inc.

The remaining authors declare that the research was conducted in the absence of any commercial or financial relationships that could be construed as a potential conflict of interest.

## Publisher’s Note

All claims expressed in this article are solely those of the authors and do not necessarily represent those of their affiliated organizations, or those of the publisher, the editors and the reviewers. Any product that may be evaluated in this article, or claim that may be made by its manufacturer, is not guaranteed or endorsed by the publisher.
