# Supplementary material for: Evidence of SARS-CoV-2-Specific T-Cell-Mediated Myocarditis in a MIS-A Case
Source: Front Immunol. 2021 Dec 9;12:779026. doi: 10.3389/fimmu.2021.779026 (PMC8695925; doi:10.3389/fimmu.2021.779026)
Supplement: Supplementary file 1 [file DataSheet_1.docx]

**Supplementary Information**

This information has been provided by the authors to give readers additional information about their work.

Supplement to: Vannella, KM et al. Evidence of SARS-CoV-2-specific T-cell-mediated myocarditis in a MIS-A case.

**Table of Contents**

**Figure S1.** Electrocardiograms………………………..…………………………………….….…3

**Figure S2.** Chest x-ray………….………………………………………..…………………….…4

**Figure S3.** Coronary angiogram………………………………………………………….…….....5

**Figure S4.** SARS-CoV-2 RNA staining (brown) of mucosal epithelium of appendix via…...…..6 RNAscope in situ hybridization

**Table S1.** SARS-CoV-2 ddPCR results by tissue…………………………………………...……7

**Table S2.** List of viruses targeted by VirCapSeq-VERT………………………………………....8

**Table S3.** Frequencies and TCRβ nucleotide sequences of the three most prevalent T-cell…....16 clonotypes amongst all T-cells in six tissues.

**Table S4.** Nucleotide sequences of the three most dominant clonotypes along with their….......17

CDR3 amino acid sequences and V and J gene hits.

**Table S5.** SARS-CoV-2 epitopes associated with the ImmuneCODE CDR3 sequences……….18 that clustered with the three most dominant clonotypes in the TCR repertoires derived

from the cardiac samples.

**Table S6.** Well-characterized SARS-CoV-2 epitopes from the immune epitope database…..…19 (IEDB) with >90% NCBI Basic Local Alignment Search Tool (BLAST) similarity to the

SARS-CoV-2 epitopes from ImmuneCODE in Table S5.

**Table S7.** Human epitopes from the immune epitope database (IEDB) with >90%....................21

NCBI Basic Local Alignment Search Tool (BLAST) similarity to the ImmuneCODE

SARS-CoV-2 epitopes in Table S5.

**Table S8.** HLA-typing………………………………………………………………………...…22

**References**…………………………………………...…………………………………………..23

**Figure S1.** Electrocardiograms. A. Hospital day 1. B. Hospital day 2.

**Figure S2.** Chest x-ray. Hospital day 1.


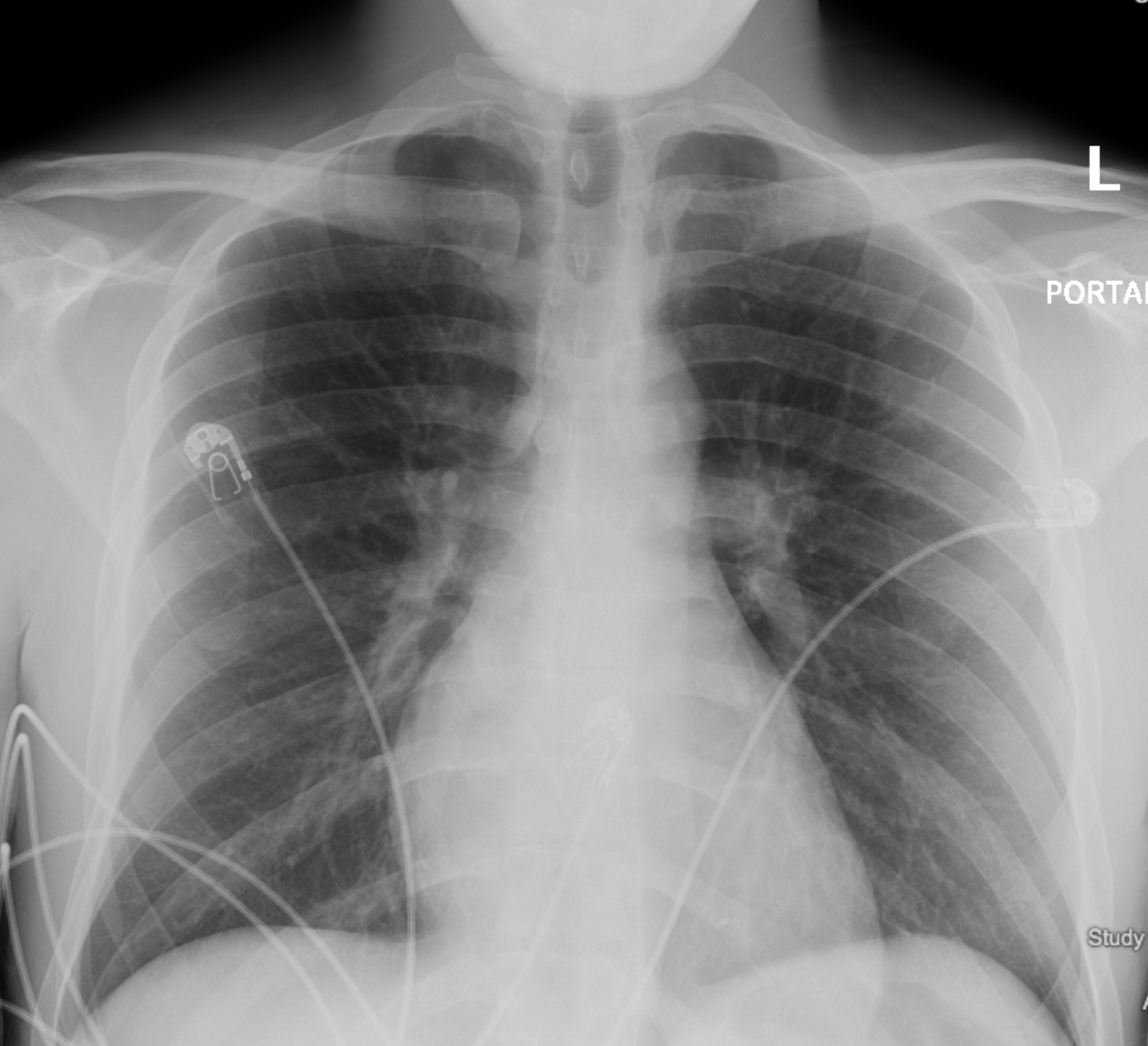


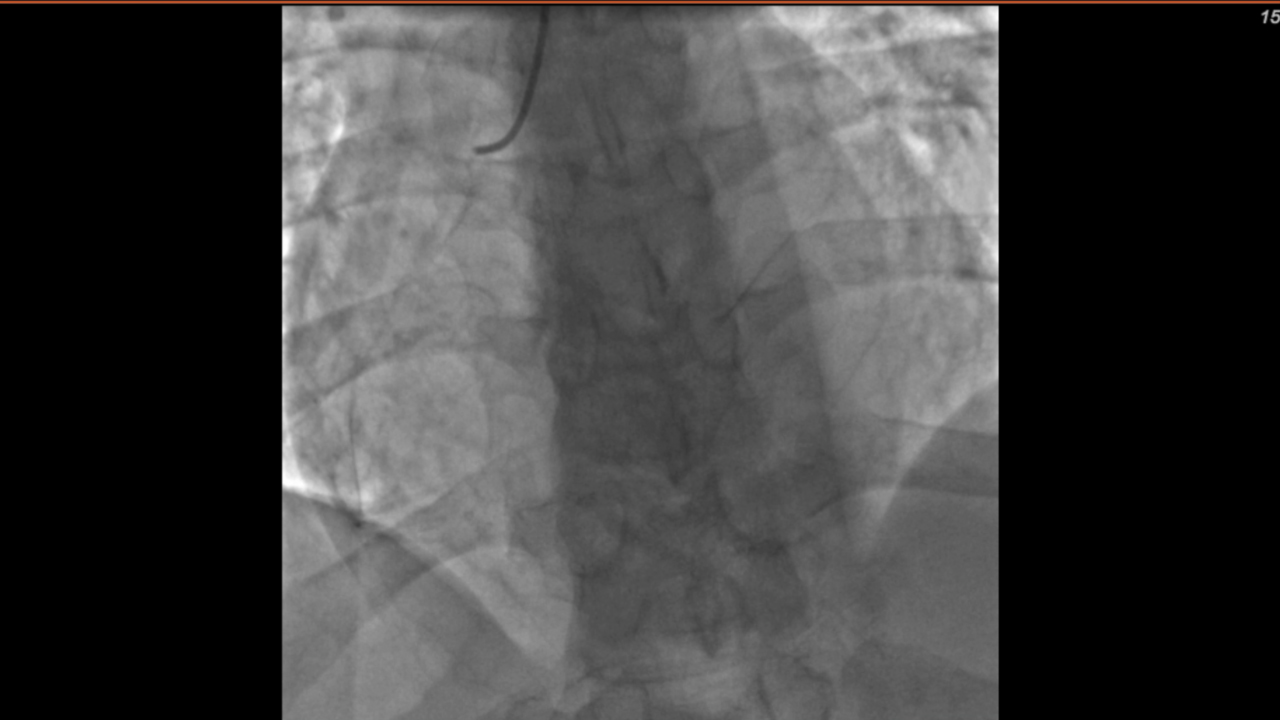

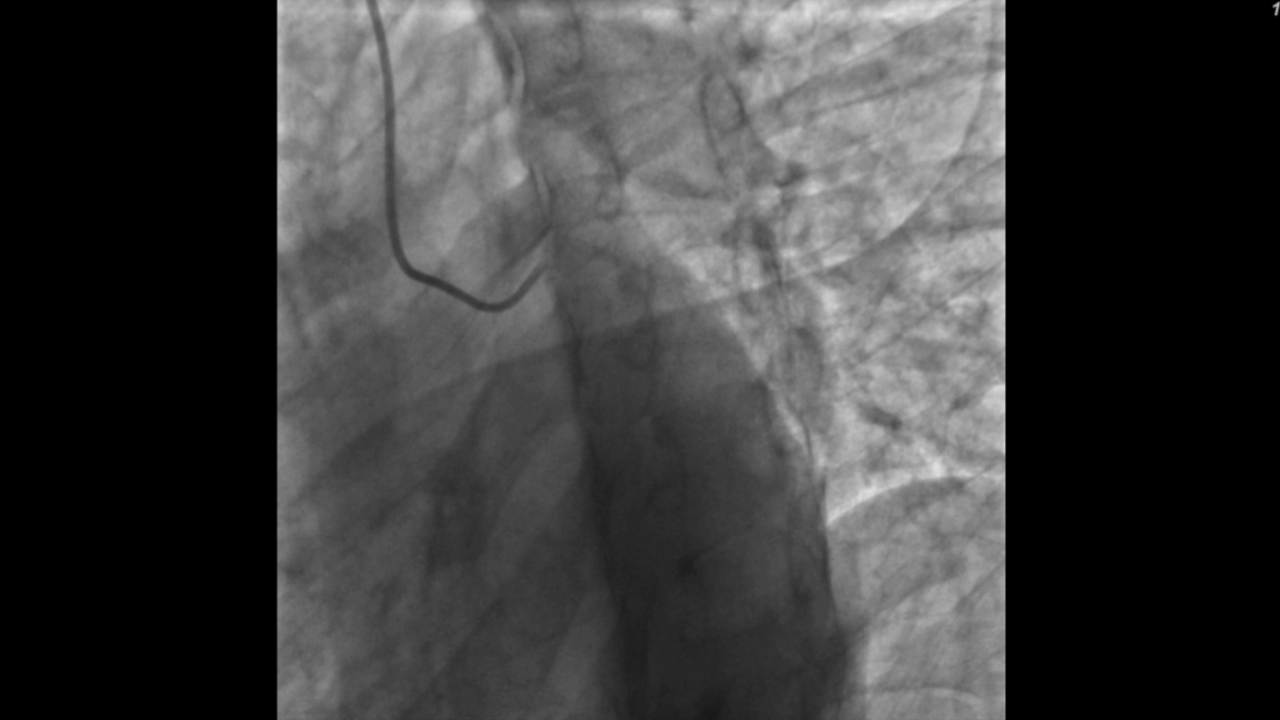
**Figure S3.** Coronary angiogram. Hospital day 2.

**Figure S4.** SARS-CoV-2 RNA staining (brown) of mucosal epithelium of appendix via RNAscope in situ hybridization. Total magnification 640X.

**Table S1.** SARS-CoV-2 ddPCR results by tissue. A tissue was considered positive (+) if it met or exceeded the limit of detection of 0.1 RNA copies/µl final ddPCR reaction and 2 positive RNA-containing droplets per ddPCR reaction for the SARS-CoV-2 nucleocapsid 1 (N1) or 2 (N2) targets. All other tissues were considered negative for SARS-CoV-2 RNA (–). The limit of detection criteria were established for respiratory swab specimens by BioRad, the manufacturer of the SARS-CoV-2 ddPCR kit. We confirmed the same lower limit of detection for tissue specimens by testing RNA extracted from 69 tissues from donors who were not previously infected with SARS-CoV-2. The 69 tissues included at least one of all the tissues assayed for this patient. All 69 tissues did not meet the criteria of 0.1 RNA copies/µl final ddPCR reaction and 2 positive RNA-containing droplets per ddPCR reaction for either the N1 or N2 target.

| **Tissue** | **N1 RNA** | **N2 RNA** |
| --- | --- | --- |
| Proximal Trachea | – | – |
| Distal Trachea | – | – |
| L Bronchus | – | – |
| R Bronchus | – | – |
| L Superior Lobe | – | – |
| L Inferior Lobe | – | – |
| R Superior Lobe | – | – |
| R Middle Lobe | – | – |
| R Inferior Lobe | – | – |
| R Ventricle | – | – |
| IV Septum | – | – |
| L Ventricle | – | – |
| Pericardium | – | – |
| Thoracic LN | – | – |
| Abdominal LN | – | – |
| Spleen | – | – |
| Appendix | – | – |
| Eyeball | – | – |
| Ileum | + | – |
| Colon | – | – |
| Liver | – | – |
| L Kidney | – | – |
| R Kidney | – | – |
| L Adrenal Gland | – | – |
| R Adrenal Gland | – | – |
| Thyroid | – | – |
| Pancreas | – | – |
| Testes | – | – |
| Skeletal Muscle | – | – |

**Table 2.** List of viruses targeted by VirCapSeq-VERT.

| **Name** | **Tax ID** |
| --- | --- |
| Adenoviridae | 10508 |
| Alloherpesviridae | 548682 |
| Alphacoronavirus | 693996 |
| Alphaherpesvirinae | 10293 |
| Alphanodavirus | 143920 |
| Alphapapillomavirus | 333750 |
| Alphapermutotetravirus | 1283211 |
| Alpharetrovirus | 153057 |
| Alphatorquevirus | 687331 |
| Alphavirus | 11019 |
| Amdoparvovirus | 310911 |
| Anelloviridae | 687329 |
| Aphthovirus | 12109 |
| Aquabirnavirus | 39750 |
| Aquamavirus | 1330065 |
| Aquaparamyxovirus | 1232658 |
| Aquareovirus | 10979 |
| Arenaviridae | 11617 |
| Arenavirus | 11618 |
| Arteriviridae | 76803 |
| Arterivirus | 11046 |
| Asfarviridae | 137992 |
| Asfivirus | 39743 |
| Astroviridae | 39733 |
| Atadenovirus | 100953 |
| Aurivirus | 1513230 |
| Avastrovirus | 249589 |
| Aveparvovirus | 1511864 |
| Aviadenovirus | 10552 |
| Avibirnavirus | 39751 |
| Avihepadnavirus | 10437 |
| Avihepatovirus | 691955 |
| Avipoxvirus | 10260 |
| Avisivirus | 1511771 |
| Avulavirus | 260963 |
| Bafinivirus | 694018 |
| Batrachovirus | 692605 |
| Betacoronavirus | 694002 |
| Betaherpesvirinae | 10357 |
| Betanodavirus | 143919 |
| Betapapillomavirus | 333922 |
| Betaretrovirus | 140052 |
| Betatorquevirus | 687332 |
| Birnaviridae | 10993 |
| Blosnavirus | 564643 |
| Bocaparvovirus | 1507401 |
| Bornaviridae | 178830 |
| Bornavirus | 186458 |
| Bracorhabdovirus | 490109 |
| Bunyaviridae | 11571 |
| Caliciviridae | 11974 |
| Capripoxvirus | 10265 |
| Cardiovirus | 12103 |
| Cervidpoxvirus | 573055 |
| Chipapillomavirus | 934800 |
| Chloriridovirus | 10491 |
| Chordopoxvirinae | 10241 |
| Circoviridae | 39724 |
| Circovirus | 39725 |
| Coltivirus | 10911 |
| Copiparvovirus | 1511888 |
| Coronaviridae | 11118 |
| Coronavirinae | 693995 |
| Cosavirus | 586418 |
| Crocodylidpoxvirus | 1285599 |
| Cuevavirus | 1513236 |
| Cyprinivirus | 692606 |
| Cytomegalovirus | 10358 |
| Cytorhabdovirus | 11305 |
| Deltacoronavirus | 1159901 |
| Deltapapillomavirus | 325454 |
| Deltaretrovirus | 153136 |
| Deltatorquevirus | 687334 |
| Deltavirus | 39759 |
| Dengue virus group | 11052 |
| Densovirinae | 40120 |
| Dependoparvovirus | 10803 |
| Dicipivirus | 1330067 |
| Dinornavirus | 674976 |
| Dyodeltapapillomavirus | 936056 |
| Dyoepsilonpapillomavirus | 935646 |
| Dyoetapapillomavirus | 935641 |
| Dyoiotapapillomavirus | 934804 |
| Dyokappapapillomavirus | 1513238 |
| Dyolambdapapillomavirus | 1513239 |
| Dyomupapillomavirus | 1513240 |
| Dyonupapillomavirus | 1513241 |
| Dyoomikronpapillomavirus | 1513242 |
| Dyopipapillomavirus | 1513243 |
| Dyorhopapillomavirus | 1513244 |
| Dyosigmapapillomavirus | 1513245 |
| Dyothetapapillomavirus | 1052159 |
| Dyoxipapillomavirus | 1513246 |
| Dyozetapapillomavirus | 934803 |
| Ebolavirus | 186536 |
| Enterovirus | 12059 |
| Entomopoxvirinae | 10284 |
| Ephemerovirus | 32613 |
| Epsilonretrovirus | 153137 |
| Epsilontorquevirus | 687335 |
| Equine lentivirus group | 11654 |
| Erbovirus | 194961 |
| Erythroparvovirus | 40121 |
| Etapapillomavirus | 325458 |
| Etatorquevirus | 687337 |
| Ferlavirus | 1283308 |
| Filoviridae | 11266 |
| Flaviviridae | 11050 |
| Flavivirus | 11051 |
| Gallivirus | 1511775 |
| Gammacoronavirus | 694013 |
| Gammaherpesvirinae | 10374 |
| Gammapapillomavirus | 325455 |
| Gammaretrovirus | 153135 |
| Gammatorquevirus | 687333 |
| Gyrovirus | 227307 |
| Hantavirus | 11598 |
| Henipavirus | 260964 |
| Hepacivirus | 11102 |
| Hepadnaviridae | 10404 |
| Hepatovirus | 12091 |
| Hepeviridae | 291484 |
| Hepevirus | 186677 |
| Herpesvirales | 548681 |
| Herpesviridae | 10292 |
| Hunnivirus | 1431456 |
| Ichtadenovirus | 691957 |
| Ictalurivirus | 172653 |
| Iltovirus | 180255 |
| Influenzavirus A | 197911 |
| Influenzavirus B | 197912 |
| Influenzavirus C | 197913 |
| Influenzavirus D | 1511083 |
| Intracisternal A-particles | 11749 |
| Iotatorquevirus | 687339 |
| Iridoviridae | 10486 |
| Iridovirus | 10487 |
| Isavirus | 324913 |
| Japanese encephalitis virus group | 11071 |
| Kappapapillomavirus | 325457 |
| Kappatorquevirus | 1218487 |
| Kobuvirus | 194960 |
| Kokobera virus group | 303179 |
| Lagovirus | 95339 |
| Lambdapapillomavirus | 325462 |
| Lambdatorquevirus | 1218489 |
| Lentivirus | 11646 |
| Leporipoxvirus | 10270 |
| Lymphocryptovirus | 10375 |
| Lymphocystivirus | 10494 |
| Lyssavirus | 11286 |
| Macavirus | 548687 |
| Malacoherpesviridae | 548685 |
| Mamastrovirus | 249588 |
| Marburgvirus | 186537 |
| Mardivirus | 180252 |
| Mastadenovirus | 10509 |
| Megalocytivirus | 308906 |
| Megrivirus | 1330069 |
| Metapneumovirus | 162387 |
| Mischivirus | 1511778 |
| Modoc virus group | 29260 |
| Molluscipoxvirus | 10278 |
| Mononegavirales | 11157 |
| Morbillivirus | 11229 |
| Mosavirus | 1481451 |
| Mosquito-borne viruses | 59562 |
| Mupapillomavirus | 334202 |
| Muromegalovirus | 10365 |
| Nairovirus | 11592 |
| Nebovirus | 696855 |
| Negevirus | 1307798 |
| Nidovirales | 76804 |
| Nodaviridae | 12283 |
| Norovirus | 142786 |
| Novirhabdovirus | 186778 |
| Ntaya virus group | 29261 |
| Nucleorhabdovirus | 11306 |
| Nupapillomavirus | 475861 |
| Nyamiviridae | 1513294 |
| Nyavirus | 1513295 |
| Omegapapillomavirus | 936061 |
| Orbivirus | 10892 |
| Orthobunyavirus | 11572 |
| Orthohepadnavirus | 10405 |
| Orthomyxoviridae | 11308 |
| Orthopoxvirus | 10242 |
| Orthoreovirus | 10882 |
| Orthoretrovirinae | 327045 |
| Oscivirus | 1511780 |
| Ostreavirus | 548686 |
| Papillomaviridae | 151340 |
| Paramyxoviridae | 11158 |
| Paramyxovirinae | 11159 |
| Parapoxvirus | 10257 |
| Parechovirus | 138954 |
| Parvoviridae | 10780 |
| Parvovirinae | 40119 |
| Pasivirus | 1511782 |
| Passerivirus | 1511802 |
| Pegivirus | 1307799 |
| Percavirus | 548688 |
| Perhabdovirus | 1298653 |
| Pestivirus | 11095 |
| Phipapillomavirus | 934802 |
| Phlebovirus | 11584 |
| Picobirnaviridae | 585893 |
| Picobirnavirus | 104394 |
| Picornavirales | 464095 |
| Picornaviridae | 12058 |
| Pipapillomavirus | 334211 |
| Pneumovirinae | 11244 |
| Pneumovirus | 11245 |
| Polyomaviridae | 151341 |
| Polyomavirus | 10624 |
| Poxviridae | 10240 |
| Proboscivirus | 548689 |
| Protoparvovirus | 1506574 |
| Psipapillomavirus | 935650 |
| Quadrivirus | 1299297 |
| Quaranjavirus | 1299308 |
| Ranavirus | 10492 |
| Recovirus | 873551 |
| Reoviridae | 10880 |
| Respirovirus | 186938 |
| Retroviridae | 11632 |
| Rhabdoviridae | 11270 |
| Rhadinovirus | 10379 |
| Rhopapillomavirus | 936057 |
| Rio Bravo virus group | 29262 |
| Rosavirus | 1511804 |
| Roseolovirus | 40272 |
| Rotavirus | 10912 |
| Rubivirus | 11040 |
| Rubulavirus | 39744 |
| Salivirus | 688449 |
| Salmonivirus | 692607 |
| Sapelovirus | 686982 |
| Sapovirus | 95341 |
| Scutavirus | 1232637 |
| Seaborne tick-borne virus group | 29264 |
| Seadornavirus | 208294 |
| Sedoreovirinae | 689832 |
| Senecavirus | 586425 |
| Siadenovirus | 129876 |
| Sigmapapillomavirus | 935635 |
| Sigmavirus | 1308858 |
| Simplexvirus | 10294 |
| Spinareovirinae | 689831 |
| Sprivivirus | 1513299 |
| Spumaretrovirinae | 327046 |
| Spumavirus | 11640 |
| Suipoxvirus | 10275 |
| Taupapillomavirus | 934799 |
| Teschovirus | 118139 |
| Tetraparvovirus | 1511911 |
| Thetapapillomavirus | 334213 |
| Thetatorquevirus | 687338 |
| Thogotovirus | 35323 |
| Tibrovirus | 1299306 |
| Tick-borne encephalitis virus group | 29263 |
| Togaviridae | 11018 |
| Torovirinae | 694017 |
| Torovirus | 11155 |
| Tremovirus | 689759 |
| Tupavirus | 1513300 |
| Upsilonpapillomavirus | 936058 |
| Varicellovirus | 10319 |
| Vesiculovirus | 11271 |
| Vesivirus | 95337 |
| Yatapoxvirus | 10282 |
| Yellow fever virus group | 40005 |
| Zetapapillomavirus | 333918 |
| Zetatorquevirus | 687336 |

**Table S3.** Frequencies and TCRβ nucleotide sequences of the three most prevalent T-cell clonotypes amongst all T cells in six tissues.

|  | **Tissue** | **Frequency** | **TCRβ sequence** |
| --- | --- | --- | --- |
| Highest frequency clonotype | Thoracic lymph node | 0.008 | ACCAGTGCCCATCCTGAAGACAGCAGCTTCTACATCTGCAGTGCCAAGGAGTGGACAGCACTTAATCAGCCCCAGCATTTTGGTGAT |
|  | Rt inferior lung lobe | 0.120 | NTGTCGGCTGCTCCCTCCCAGACATCTGTGTACTTCTGTGCCAGCAGTTACTCCAGCGGATTTACAGATACGCAGTATTTTGGCCCA |
|  | Lt ventricle | 0.567 | NTGTCGGCTGCTCCCTCCCAGACATCTGTGTACTTCTGTGCCAGCAGTTACTCCAGCGGATTTACAGATACGCAGTATTTTGGCCCA |
|  | Rt ventricle | 0.268 | NTGTCGGCTGCTCCCTCCCAGACATCTGTGTACTTCTGTGCCAGCAGTTACTCCAGCGGATTTACAGATACGCAGTATTTTGGCCCA |
|  | Interventricular septum | 0.586 | NTGTCGGCTGCTCCCTCCCAGACATCTGTGTACTTCTGTGCCAGCAGTTACTCCAGCGGATTTACAGATACGCAGTATTTTGGCCCA |
|  | Pericardium | 0.048 | NTGTCGGCTGCTCCCTCCCAGACATCTGTGTACTTCTGTGCCAGCAGTTACTCCAGCGGATTTACAGATACGCAGTATTTTGGCCCA |
| Second highest frequency clonotype | Thoracic lymph node | 0.005 | ACGTTGGCGTCTGCTGTACCCTCTCAGACATCTGTGTACTTCTGTGCCAGCAGTGACTCGAGTGAGGGTGAGCAGTTCTTCGGGCCA |
|  | Rt inferior lung lobe | 0.037 | GCCCTGCAGCCAGAAGACTCAGCCCTGTATCTCTGCGCCAGCAGCCAAGATAGAGCGGGGGGACTAGGAGAGCAGTACTTCGGGCCG |
|  | Lt ventricle | 0.043 | GTGAACGCCTTGGAGCTGGACGACTCGGCCCTGTATCTCTGTGCCAGCAGCCGGCTAGCGGGGCGAGAGACCCAGTACTTCGGGCCA |
|  | Rt ventricle | 0.025 | GTGAACGCCTTGGAGCTGGACGACTCGGCCCTGTATCTCTGTGCCAGCAGCCGGCTAGCGGGGCGAGAGACCCAGTACTTCGGGCCA |
|  | Interventricular septum | 0.040 | GTGAACGCCTTGGAGCTGGACGACTCGGCCCTGTATCTCTGTGCCAGCAGCCGGCTAGCGGGGCGAGAGACCCAGTACTTCGGGCCA |
|  | Pericardium | 0.022 | ATCCAGCGCACAGAGCAGGGGGACTCGGCCATGTATCTCTGTGCCAGCAGCTCGATCCGTATAAACACTGAAGCTTTCTTTGGACAA |
| Third highest frequency clonotype | Thoracic lymph node | 0.004 | NTGTCGGCTGCTCCCTCCCAGACATCTGTGTACTTCTGTGCCAGCAGTTACTCCAGCGGATTTACAGATACGCAGTATTTTGGCCCA |
|  | Rt inferior lung lobe | 0.028 | ATCCAGTCCACGGAGTCAGGGGACACAGCACTGTATTTCTGTGCCAGCAGCAAAGTTGGGGGAACAGATACGCAGTATTTTGGCCCA |
|  | Lt ventricle | 0.014 | CTGGAGTCAGCTACCCGCTCCCAGACATCTGTGTATTTCTGCGCCAGCAGCGACAGTCTCCTCAATCAGCCCCAGCATTTTGGTGAT |
|  | Rt ventricle | 0.010 | CTGGAGTCAGCTACCCGCTCCCAGACATCTGTGTATTTCTGCGCCAGCAGCGACAGTCTCCTCAATCAGCCCCAGCATTTTGGTGAT |
|  | Interventricular septum | 0.017 | CTGGAGTCAGCTACCCGCTCCCAGACATCTGTGTATTTCTGCGCCAGCAGCGACAGTCTCCTCAATCAGCCCCAGCATTTTGGTGAT |
|  | Pericardium | 0.019 | GCCCTGCAGCCAGAAGACTCAGCCCTGTATCTCTGCGCCAGCAGCCAAGATAGAGCGGGGGGACTAGGAGAGCAGTACTTCGGGCCG |

**Table S4.** Nucleotide sequences of the three most dominant clonotypes along with their CDR3 amino acid sequences and V and J gene hits. Clonotypes corresponding to each clone were defined by the CDR3 amino acid sequence resulting from the alignment of the clone's nucleotide sequence against the human VDJ reference. This reference includes the germline nucleotide sequences for the human V, D, and J genes. We used MiXCR^1^ to confirm the CDR3 amino acid sequence and V and J gene hits identified by the Adaptive Biotech pipeline, and also to check for additional gene hits that had alignment scores (https://mixcr.readthedocs.io/en/latest/align.html) within 10% of the top gene hit for each clone.

| **Clonotype** | **Full nucleotide sequence** | **CDR3 nucleotide sequence** | **CDR3 amino acid sequence (clonotype)** | **V gene hits (alignment score)** | **J gene hits (alignment score)** |
| --- | --- | --- | --- | --- | --- |
| 1 | NTGTCGGCTGCTCCCTCCCAGACATCTGTGTACTTCTGTGCCAGCAGTTACTCCAGCGGATTTACAGATACGCAGTATTTTGGCCCA | TGTGCCAGCAGTTACTCCAGCGGATTTACAGATACGCAGTATTTT | CASSYSSGFTDTQYF | TRBV6-5 (261),TRBV6-8 (247),TRBV6-2 (242),TRBV6-3 (242),TRBV6-6 (242) | TRBJ2-3 (120) |
| 2 | GTGAACGCCTTGGAGCTGGACGACTCGGCCCTGTATCTCTGTGCCAGCAGCCGGCTAGCGGGGCGAGAGACCCAGTACTTCGGGCCA | TGTGCCAGCAGCCGGCTAGCGGGGCGAGAGACCCAGTACTTC | CASSRLAGRETQYF | TRBV5-4 (255),TRBV5-8 (241) | TRBJ2-5 (110) |
| 3 | CTGGAGTCAGCTACCCGCTCCCAGACATCTGTGTATTTCTGCGCCAGCAGCGACAGTCTCCTCAATCAGCCCCAGCATTTTGGTGAT | TGCGCCAGCAGCGACAGTCTCCTCAATCAGCCCCAGCATTTT | CASSDSLLNQPQHF | TRBV10-2 (251) | TRBJ1-5 (125) |

**Table S5.** SARS-CoV-2 epitopes associated with the ImmuneCODE CDR3 sequences that clustered with the three most dominant clonotypes in the TCR repertoires derived from the cardiac samples. The clustering threshold is 90% global sequence identity, defined by CD-HIT^2^ as the number of identical amino acids in alignment divided by the total number of amino acids in the shorter sequence, with an allowed sequence length difference of up to two amino acids per alignment. The listed ImmuneCODE clonotypes have >90% global sequence identity matching with the three most dominant clonotypes at the amino acid sequence level.

| **Clonotype** | **ImmuneCODE clonotype** | **SARS-CoV-2 antigen** | **SARS-CoV-2 epitopes** |
| --- | --- | --- | --- |
| 1 | CASSYSSGGTDTQYF+TCRBV06-06+TCRBJ02-03 | ORF1ab | AEAELAKNVSL,AELAKNVSLDNVL |
| 1 | CASSYSTFTDTQYF+TCRBV06-02/06-03+TCRBJ02-03 | surface glycoprotein | FPQSAPHGV,FPQSAPHGVVF |
| 1 | CASSYSSVTDTQYF+TCRBV06-02/06-03+TCRBJ02-03 | surface glycoprotein | FPQSAPHGV,FPQSAPHGVVF |
| 1 | CASSYSSPTDTQYF+TCRBV06-05+TCRBJ02-03 | ORF7b | AFLLFLVLI,FLAFLLFLV,FYLCFLAFL,FYLCFLAFLL,IDFYLCFLAF,IELSLIDFYL,LIDFYLCFL,LLFLVLIML,MIELSLIDFY,SLIDFYLCFL,YLCFLAFLL |
| 1 | CASSYSPGFTDTQYF+TCRBV06-05+TCRBJ02-03 | ORF7b | AFLLFLVLI,FLAFLLFLV,FYLCFLAFL,FYLCFLAFLL,IDFYLCFLAF,IELSLIDFYL,LIDFYLCFL,LLFLVLIML,MIELSLIDFY,SLIDFYLCFL,YLCFLAFLL |
| 2 | CASSRLAGREQYF+TCRBV06-02/06-03+TCRBJ02-07 | ORF10 | AFPFTIYSL,GYINVFAFPF,INVFAFPFTI,MGYINVFAF,NVFAFPFTI,NVFAFPFTIY,YINVFAFPF |
| 3 | CASSDSLNQPQHF+TCRBV02-01+TCRBJ01-05 | ORF1ab | VLWAHGFEL |
| 3 | CASSLLNQPQHF+TCRBV07-08+TCRBJ01-05 | ORF7b | AFLLFLVLI,FLAFLLFLV,FYLCFLAFL,FYLCFLAFLL,IDFYLCFLAF,IELSLIDFYL,LIDFYLCFL,LLFLVLIML,MIELSLIDFY,SLIDFYLCFL,YLCFLAFLL |

**Table S6.** Well-characterized SARS-CoV-2 epitopes from the immune epitope database (IEDB)^3^ with >90% NCBI Basic Local Alignment Search Tool (BLAST) similarity to the SARS-CoV-2 epitopes from ImmuneCODE in Table S5. The last column lists the epitope-specific HLA restrictions^4^ that closely match the HLA typing data from the patient.

| **Associated clonotype** | **Epitope ID** | **Object Type** | **Description** | **Starting Position** | **Ending Position** | **Antigen Name** | **Antigen Accession** | **Parent Protein** | **Parent Protein Accession** | **Organism** | **HLA restrictions** |
| --- | --- | --- | --- | --- | --- | --- | --- | --- | --- | --- | --- |
| 1 | 1074839 | Linear peptide | AELAKNVSLDNVL | 2618 | 2630 | orf1ab polyprotein | YP_009724389.1 | Replicase polyprotein 1ab | P0DTD1 | SARS-CoV-2 |  |
| 1 | 1312109 | Linear peptide | AELAKNVSL | 2618 | 2626 | orf1ab polyprotein | QHD43415.1 | Replicase polyprotein 1ab | P0DTD1 | SARS-CoV-2 | HLA-B*44:02 |
| 1 | 1314190 | Linear peptide | AEAELAKNV | 2616 | 2624 | orf1ab polyprotein | YP_009724389.1 | Replicase polyprotein 1ab | P0DTD1 | SARS-CoV-2 | HLA-B*44:02\|HLA-B*44:03 |
| 1 | 1324559 | Linear peptide | SLDNVLSTF | 2625 | 2633 | orf1ab polyprotein | YP_009724389.1 | Replicase polyprotein 1ab | P0DTD1 | SARS-CoV-2 |  |
| 1 | 1072604 | Linear peptide | SFPQSAPHGVVFLHV | 1051 | 1065 | surface glycoprotein | QHD43416.1 | Spike glycoprotein | P0DTC2 | SARS-CoV-2 |  |
| 1 | 1074891 | Linear peptide | FPQSAPHGVVF | 1052 | 1062 | surface glycoprotein | YP_009724390.1 | Spike glycoprotein | P0DTC2 | SARS-CoV-2 |  |
| 1 | 1075023 | Linear peptide | QSAPHGVVFLHVTYVPAQEKNFTTAPAICHDGKAHFPREGVFV | 1054 | 1096 | surface glycoprotein | YP_009724390.1 | Spike glycoprotein | P0DTC2 | SARS-CoV-2 |  |
| 1 | 1316853 | Linear peptide | FPQSAPHGV | 1052 | 1060 | surface glycoprotein | YP_009724390.1 | Spike glycoprotein | P0DTC2 | SARS-CoV-2 |  |
| 1 | 1324414 | Linear peptide | SFPQSAPHGVVF | 1051 | 1062 | surface glycoprotein | YP_009724390.1 | Spike glycoprotein | P0DTC2 | SARS-CoV-2 |  |
| 1 | 1333222 | Linear peptide | QSAPHGVVF | 1054 | 1062 | surface glycoprotein | YP_009724390.1 | Spike glycoprotein | P0DTC2 | SARS-CoV-2 | HLA-C*07:02 |
| 1 | 3589 | Linear peptide | APHGVVFLHV | 1056 | 1065 | surface glycoprotein | YP_009724390.1 | Spike glycoprotein | P0DTC2 | SARS-CoV-2 |  |
| 1 | 1071768 | Linear peptide | PHGVVFLHVTYVPAQ | 1057 | 1071 | surface glycoprotein | QHD43416.1 | Spike glycoprotein | P0DTC2 | SARS-CoV-2 |  |
| 1 | 1074847 | Linear peptide | APHGVVFLHVTYV | 1056 | 1068 | surface glycoprotein | YP_009724390.1 | Spike glycoprotein | P0DTC2 | SARS-CoV-2 |  |
| 1 | 1309428 | Linear peptide | APHGVVFLHVTYVPAQEKNF | 1056 | 1075 | S protein | QII57161.1 | Spike glycoprotein | P0DTC2 | SARS-CoV-2 |  |
| 1 | 1310281 | Linear peptide | APHGVVFLHVTYVPA | 1056 | 1070 | surface glycoprotein | QKE11719.1 | Spike glycoprotein | P0DTC2 | SARS-CoV-2 |  |
| 1 | 1330420 | Linear peptide | APHGVVFL | 1056 | 1063 | surface glycoprotein | YP_009724390.1 | Spike glycoprotein | P0DTC2 | SARS-CoV-2 |  |
| 1 | 1074999 | Linear peptide | MIELSLIDFYLCFLAFLLFLVLIML | 1 | 25 | ORF7b | YP_009725296.1 | ORF7b protein | P0DTD8 | SARS-CoV-2 |  |
| 1 | 1331247 | Linear peptide | DFYLCFLAFLLFLVL | 8 | 22 | ORF7b, partial | QKG90752.1 | ORF7b protein | P0DTD8 | SARS-CoV-2 |  |
| 2 | 1074996 | Linear peptide | MGYINVFAFPFTIYSL | 1 | 16 | ORF10 protein | YP_009725255.1 | ORF10 protein | A0A663DJA2 | SARS-CoV-2 |  |
| 2 | 1074997 | Linear peptide | MGYINVFAFPFTIYSLLLCRMN | 1 | 22 | ORF10 protein | YP_009725255.1 | ORF10 protein | A0A663DJA2 | SARS-CoV-2 |  |
| 2 | 1310502 | Linear peptide | INVFAFPFTIYSLLL | 4 | 18 | ORF10 protein | QHI42199.1 | ORF10 protein | A0A663DJA2 | SARS-CoV-2 |  |
| 2 | 1330542 | Linear peptide | NVFAFPFTI | 5 | 13 | ORF10 protein | QHI42199.1 | ORF10 protein | A0A663DJA2 | SARS-CoV-2 | HLA-A*02:01 |
| 2 | 1334359 | Linear peptide | YINVFAFPF | 3 | 11 | ORF10 protein | QHI42199.1 | ORF10 protein | A0A663DJA2 | SARS-CoV-2 | HLA-A*02:01 |
| 3 | 69850 | Linear peptide | VLWAHGFEL | 6109 | 6117 | orf1ab polyprotein | YP_009724389.1 | Replicase polyprotein 1ab | P0DTD1 | SARS-CoV-2 | HLA-A*02:01\|HLA-B*15:01 |
| 3 | 1074999 | Linear peptide | MIELSLIDFYLCFLAFLLFLVLIML | 1 | 25 | ORF7b | YP_009725296.1 | ORF7b protein | P0DTD8 | SARS-CoV-2 |  |
| 3 | 1331247 | Linear peptide | DFYLCFLAFLLFLVL | 8 | 22 | ORF7b, partial | QKG90752.1 | ORF7b protein | P0DTD8 | SARS-CoV-2 |  |

**Table S7.** Human epitopes from the immune epitope database (IEDB)^3^ with >90% NCBI Basic Local Alignment Search Tool (BLAST) similarity to the ImmuneCODE SARS-CoV-2 epitopes in Table S5.

| **Associated clonotypes** | **Epitope ID** | **Object Type** | **Description** | **Starting Position** | **Ending Position** | **Antigen Name** | **Antigen Accession** | **Parent Protein** | **Parent Protein Accession** | **Organism** |
| --- | --- | --- | --- | --- | --- | --- | --- | --- | --- | --- |
| 1 | 707855 | Linear peptide | GPFAKNVSL | 1095 | 1103 | DNA polymerase theta | NP_955452.3 | DNA polymerase theta | O75417 | Homo sapiens |
| 1 | 774688 | Linear peptide | KRQELEAELAK | 1735 | 1745 | plectin isoform 1f | NP_958780.1 | Plectin | Q15149 | Homo sapiens |
| 1 | 1006938 | Linear peptide | EAELAKVRAEMEVLLA | 1891 | 1906 | Plectin | Q15149 | Plectin | Q15149 | Homo sapiens |
| 1 | 1247861 | Linear peptide | LEAELAKV | 1890 | 1897 | Plectin | Q15149 | Plectin | Q15149 | Homo sapiens |
| 1 and 3 | 506412 | Linear peptide | KLFLVLII | 2 | 9 | Beta-defensin 128 | Q7Z7B8.1 | Beta-defensin 128 | Q7Z7B8 | Homo sapiens |
| 1 and 3 | 708283 | Linear peptide | GRPLFLVLI | 613 | 621 | Phosphorylase b kinase regulatory subunit beta | Q93100.3 | Phosphorylase b kinase regulatory subunit beta | Q93100 | Homo sapiens |
| 1 and 3 | 734196 | Linear peptide | EHDIELSLI | 268 | 276 | RUN and TBC1 domain containing 3, isoform CRA_b | EAW60379.1 | Small G protein signaling modulator 3 | Q96HU1 | Homo sapiens |

**Table S8.** HLA-typing.

| A* | A* | B* | B* | C* | C* | DRB1* | DRB1* | DQB1* | DQB1* | DRB3* | DQA1* | DQA1* | DPB1* | DPB1* |
| --- | --- | --- | --- | --- | --- | --- | --- | --- | --- | --- | --- | --- | --- | --- |
| 02:01:01 | 66:01:01 | 13:02:01 | 42:01:01 | 06:02:01 | 17:01:01 | 01:01:01/01:100 | 03:01:01/03:147 | 02:01:01 | 05:01:01 | 02:02:01 | 01:01:01 | 05:01:01 | 02:01:02 | 105:01:01/665:01 |

**References**

1. Bolotin DA, Poslavsky S, Mitrophanov I, et al. MiXCR: software for comprehensive adaptive immunity profiling. Nat Methods 2015;12:380-1.

2. Fu L, Niu B, Zhu Z, Wu S, Li W. CD-HIT: accelerated for clustering the next-generation sequencing data. Bioinformatics 2012;28:3150-2.

3. Vita R, Mahajan S, Overton JA, et al. The Immune Epitope Database (IEDB): 2018 update. Nucleic Acids Res 2019;47:D339-D43.

4. Tarke A, Sidney J, Kidd CK, et al. Comprehensive analysis of T cell immunodominance and immunoprevalence of SARS-CoV-2 epitopes in COVID-19 cases. Cell Rep Med 2021;2:100204.
